# Supplementary material for: The hypoxia response pathway promotes PEP carboxykinase and gluconeogenesis in C. elegans
Source: Nat Commun. 2022 Oct 18;13:6168. doi: 10.1038/s41467-022-33849-x (PMC9579151; doi:10.1038/s41467-022-33849-x)
Supplement: Supplementary file 1 — Supplementary Information [file 41467_2022_33849_MOESM1_ESM.pdf]

SUPPLEMENTARY INFORMATION

Title: The Hypoxia Response Pathway Promotes PEP Carboxykinase And Gluconeogenesis In *C. elegans*

Authors: Mehul Vora, Stephanie M. Pyonteck, Tatiana Popovitchenko, Tarmie L. Matlack, Aparna Prashar, Nanci S. Kane, John Favate, Premal Shah, and Christopher Rongo

SUPPLEMENTARY FIGURE 1 2
SUPPLEMENTARY FIGURE 2 4
SUPPLEMENTARY FIGURE 3 6
SUPPLEMENTARY FIGURE 4 8
SUPPLEMENTARY FIGURE 5 10
SUPPLEMENTARY FIGURE 6 12
SUPPLEMENTARY FIGURE 7 14
SUPPLEMENTARY FIGURE 8 16
SUPPLEMENTARY FIGURE 9 18

Supplementary Figure 1

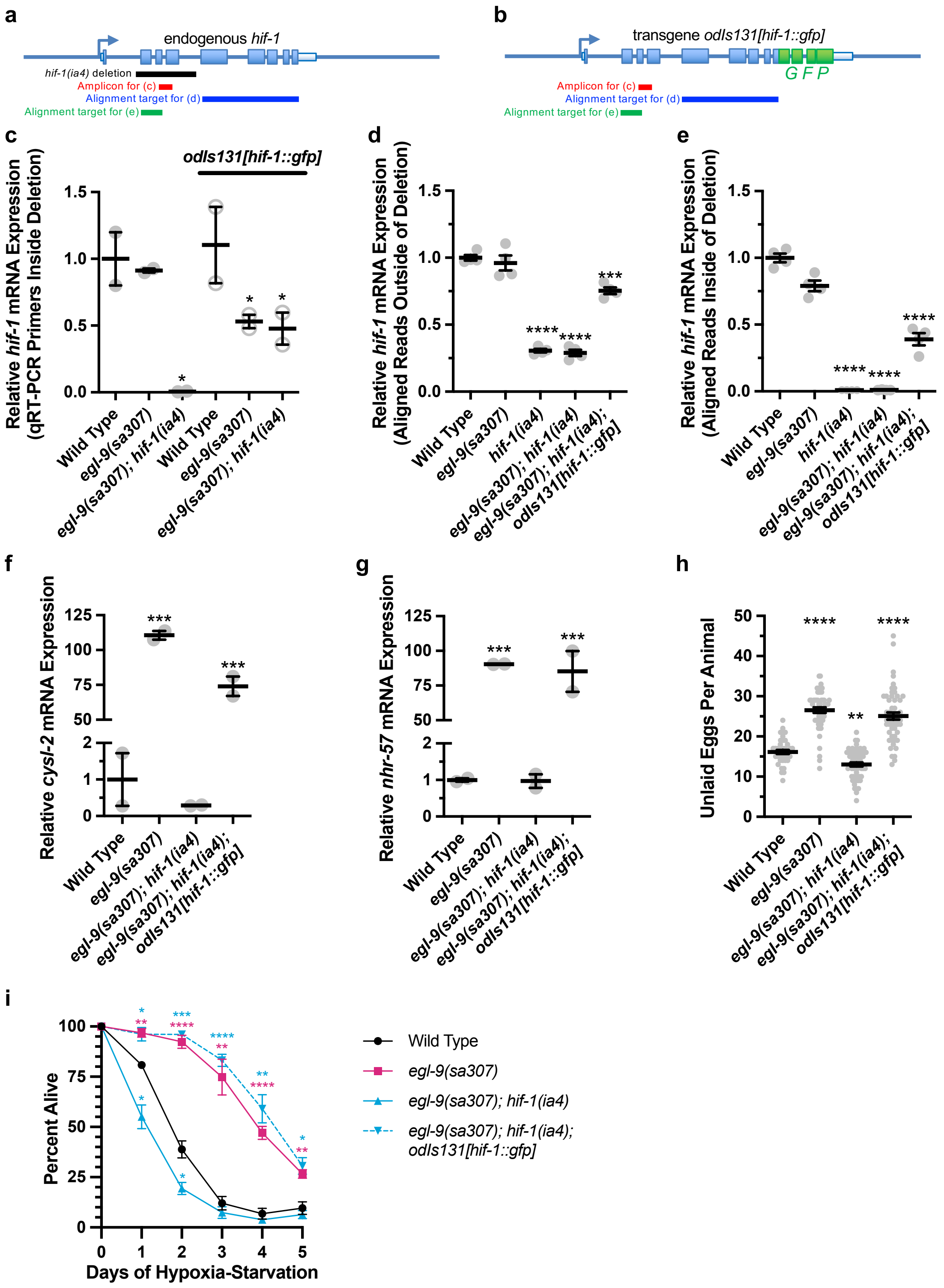

Supplementary Figure 1 Legend. The *odIs131[hif-1::gfp]* transgene behaves like endogenous *hif-1*.

Diagrams of the (a) *hif-1* locus and the (b) *odIs131[hif-1::gfp]* transgene. Arrows indicate TSS. Boxes indicate exons. Black bar indicates sequences deleted in *hif-1(ia4)*. Red bar indicates qRT-PCR amplicon in (c). Blue and green bars indicate the sequence targets to which RNA-seq reads were aligned in (d) and (e), respectively. (c-e) Relative *hif-1* mRNA expression levels determined by (c) qRT-PCR or (d,e) RNA-seq read counts per total million reads in the indicated background. Primers in (c) anneal to sequences inside the *hif-1(ia4)* deletion and thus only detect transgenic *hif-1* transcripts in the *egl-9(sa307); hif-1(ia4); odIs131[hif-1::gfp]* background. RNA-seq reads were aligned to (d) exons 5-9 outside of the deletion (thus measuring both wild-type transcripts and transcripts containing the deletion), or (e) exons 2-3 inside of the deletion (thus measuring only wild-type transcripts). The *odIs131[hif-1::gfp]* transgene inserted on the X chromosome; its expression level is consistent with X-linked dosage compensation. All expression is normalized to wild type. (f,g) Relative mRNA levels for the indicated HIF-1 target genes in the indicated genotypes determined by qRT-PCR. (h) Average number of unlaid eggs *in utero* per animal for the indicated genotype (n=40-55 animals). (i) Average percent of animals alive on the given day of exposure to hypoxia without food (n=5 trials, 50-120 animals per trial, starting with L4 stage as day 0). All other experiments were under normoxia. Results in (d-g) demonstrate that the *odIs131[hif-1::gfp]* transgene is sufficient to conduct HIF-1 physiological functions when expressed at the level of endogenous *hif-1*, which is expected given that all reported *hif-1* mutant phenotypes are recessive. Error bars indicate mean ± SEM. Number of biological replicates for (c), (d), (e), (f), and (g) were 2, 4, 4, 2, and 2, respectively. For (h), the number of animals scored for egg laying were 40 for wild type, 51 for *egl-9(sa307)*, 55 for *egl-9(sa307) hif-1(ia4)*, and 54 for *egl-9 hif-1* rescued with *odIs131*. ****$p<0.0001$, ***$p<0.001$, **$p<0.01$, *$p<0.05$ compared to wild type using ANOVA/Dunnett's multiple comparison two-sided test.

Supplementary Figure 2

a

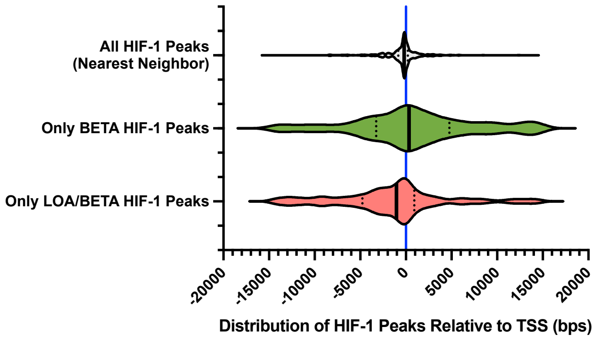

b

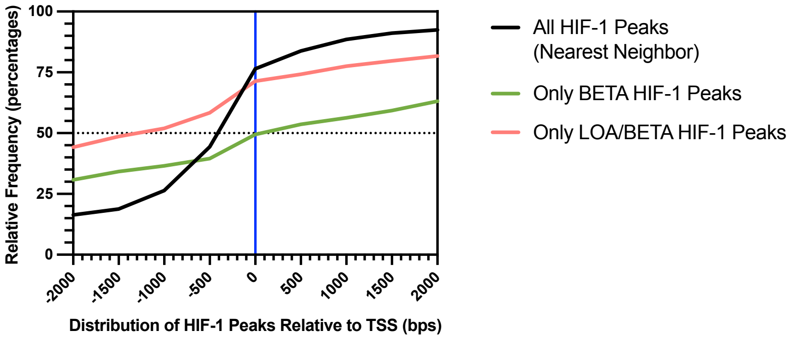

c

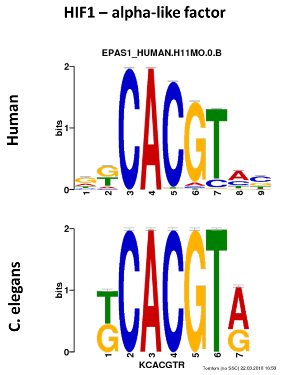

Supplementary Figure 2 Legend. HIF-1 binding sites contain HRE sequences and are located near genes.

(a) Violin plot of the number of HIF-1 binding sites (ChIP-seq peaks) against the distance of those peaks from the TSS of either the nearest genes to all HIF-1 peaks (gray), the target genes identified by BETA analysis (green), or the target genes identified by LOA combined with BETA analysis (red). (b) Cumulative probability distribution of the HIF-1 bindings site, as per (a), focused around to 2 kb up and downstream of the TSS (indicated by the vertical blue line at point 0 on the x-axis). Targets identified with the nearest-neighbor approach cluster within about 500 bps of the TSS, whereas BETA or the combination of LOA and BETA identify more distal targets within about 15 kb range of the TSS. The nearest neighbor and LOA/BETA analysis showed a bias towards upstream sites. Vertical solid lines in the violin plots indicate mean, whereas vertical dotted lines indicate quartiles. Horizontal dashed line in (b) indicates the midpoint of the frequency distribution for reference. (c) Consensus HRE sequences identified by MEME-Suite in humans and enriched in all HIF-1 ChIP-seq peaks in *C. elegans* ChIP-seq sequences.

Supplementary Figure 3

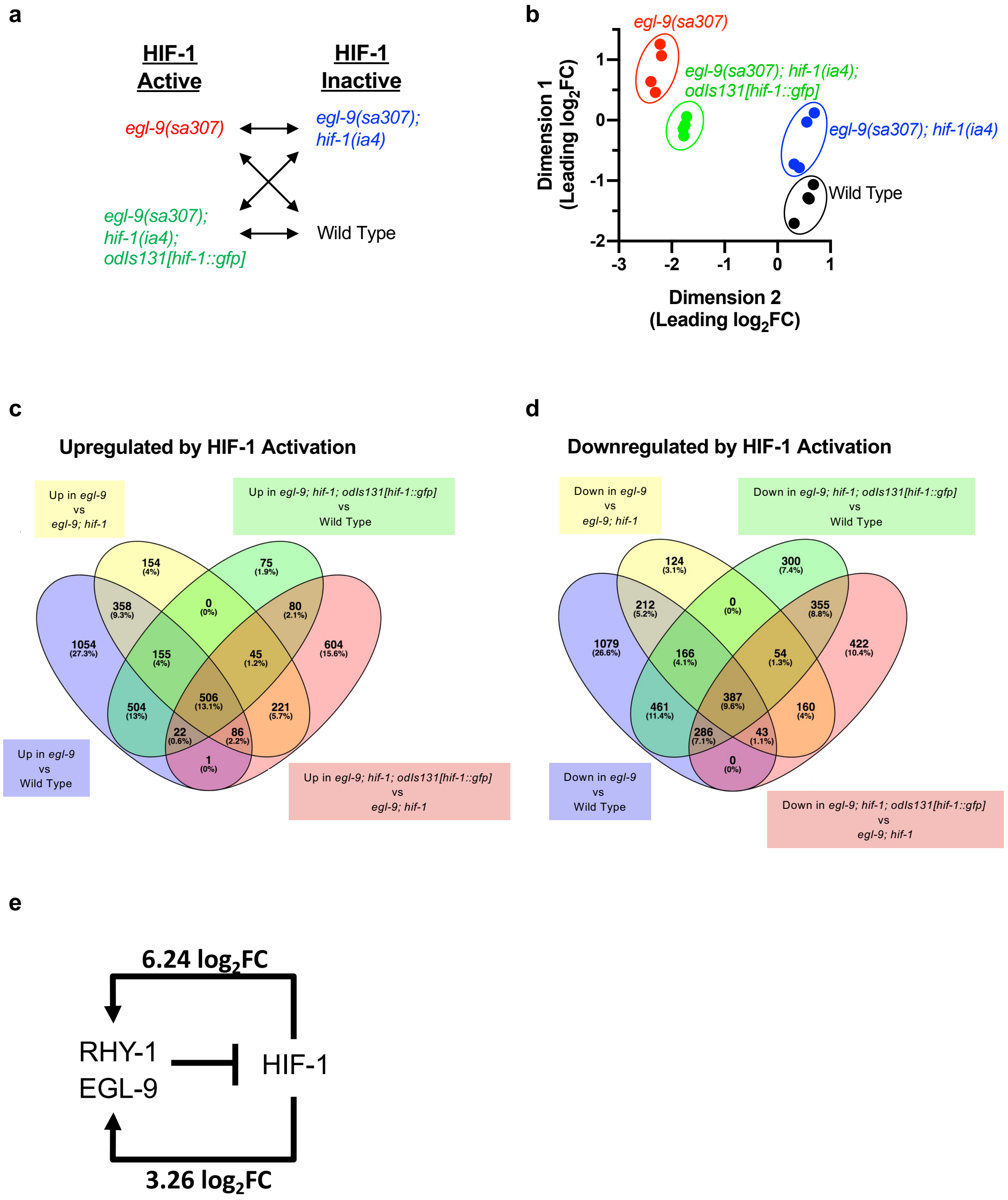

Supplementary Figure 3 Legend. RNA-seq of several genotypes identifies high-confidence targets.

(a) The four different pairwise comparisons of RNA-seq expression data used to identify genes whose expression is changed when HIF-1 is active versus inactive under aerobic conditions.
(b) Multidimensional Scaling analysis of RNA-seq gene expression data for four replicates each of four different genotypes. (c-d) Venn diagrams of the number (and percentage of total) of genes with significant differential expression in the indicated pairwise comparison. (c) Genes that are upregulated when HIF-1 is active. (d) Genes with downregulated expression when HIF-1 is active. (e) The genes *egl-9* and *rhy-1*, which are negative regulators of HIF-1, are some of the strongest upregulated targets of HIF-1, highlighting the importance of negative feedback regulation in the pathway.

Supplementary Figure 4

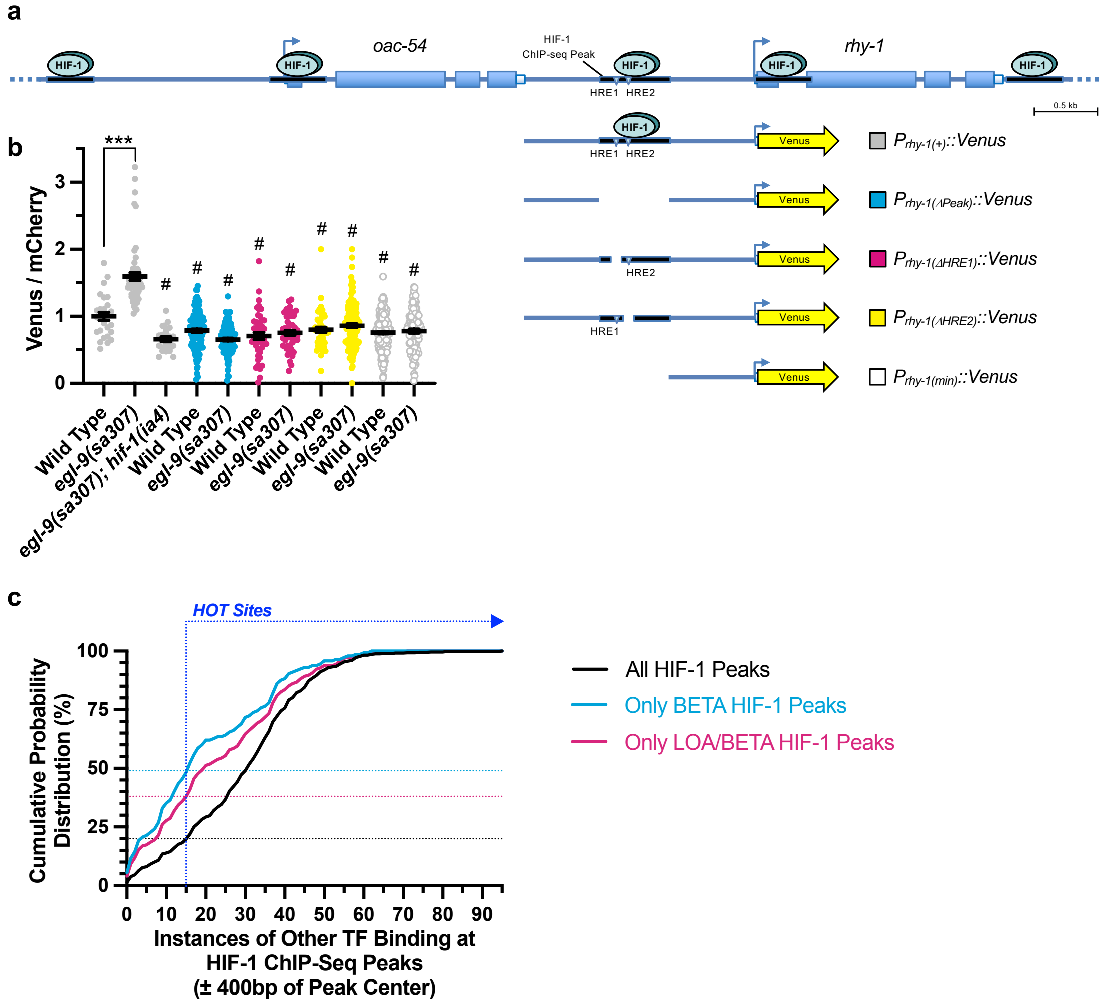

Supplementary Figure 4 Legend. Validation of HIF-1 target genes identified by LOA and BETA.

(a) Cartoon of the *rhy-1* locus and the different Venus-based promoters used to examine its regulated expression. Boxes indicate exons. Arrows indicate the TSS. The ovals and black lines beneath them indicate HIF-1 and the HIF-1 binding site identified by ChIP-seq, respectively. The inverted triangles indicate the sites of the HRE motif. The yellow arrow indicates sequences encoding the fluorescent Venus reporter. The *oac-54* gene is nearby this cluster of HIF-1 binding sites and also shows HIF-1-dependent regulation. (b) Graph of Venus/mCherry fluorescence ratios for the indicated genotype under normoxia. Dot color indicates specific reporter as per legend on right side of panel A. Error bars indicate mean ± SEM. ***$p<0.001$ ANOVA/Dunnett's Multiple Comparison two-sided test compared to wild type. #$p<0.001$ ANOVA/Dunnett's Multiple Comparison two-sided test compared to *egl-9(sa307)*. The number of animals scored for fluorescence for each column (left to right): 27, 59, 24, 118, 97, 46, 47, 47, 142, 189, and 106. (c) Cumulative probability distribution measuring the number of HIF-1 binding sites (ChIP-seq peaks) against the number of other transcription factors known to bind to each site's region of the genome (within 400 bps of each ChIP-seq peak center). The black line indicates all HIF-1 binding sites, whereas the cyan and magenta lines indicate HIF-1 binding sites identified by BETA analysis alone or a combination of LOA and BETA, respectively. The blue dotted line indicates high occupancy target (HOT) sites determined by modENCODE. The black dotted line indicates the point in the probability distribution (20%) at which all HIF-1 peaks begin to overlap with HOT binding sites for 15 or more TFs. The cyan dotted line indicates the point in the probability distribution (49%) at which HIF-1 peaks identified by BETA analysis only begin to overlap with HOT sites. The magenta dotted line shows a similar point (38%) for only LOA/BETA HIF-1 peaks.

Supplementary Figure 5

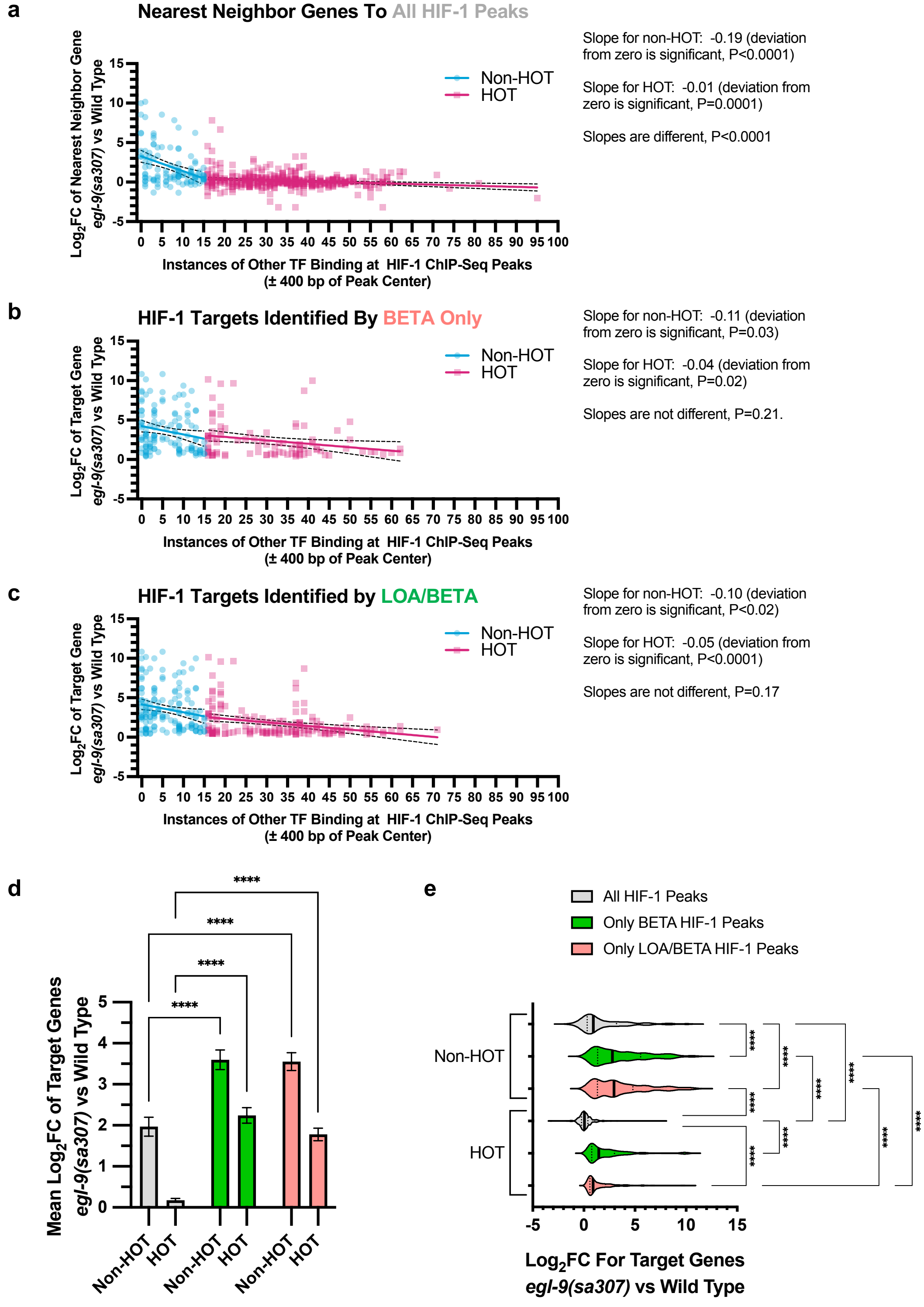

Supplementary Figure 5 Legend. LOA and BETA Can Distinguish Functional From Non-functional HOT sites.

(a-c) Graphs of log_2 fold change (plotted on the ordinate) in mRNA levels in *egl-9(sa307)* mutants versus wild type for individual target genes (dots) associated with individual HIF-1 binding sites (ChIP-seq peaks). Cyan dots are target genes associated with a non-HOT site HIF-1 ChIP-seq peak (i.e., a site at which fewer than 15 other transcription factors (TFs) are known to bind). Magenta dots are target genes associated with a HOT site HIF-1 ChIP-seq peak (i.e., a site at which 15 or more other TFs are known to bind). The abscissa indicates the number of TFs for each site. Linear regression was performed separately for HOT sites and non-HOT sites, with slopes and statistical parameters indicated to the right of each graph. Dotted lines flanking the curve fit indicate the 95% confidence interval. Target genes were identified as either (a) nearest neighbor gene for all HIF-1 binding sites (peaks), (b) target genes identified by the BETA algorithm only, or (c) target genes identified by a combination of BETA and LOA. (d) Graph of the mean differential regulation observed for target genes identified as nearest neighbor to all sites (gray bars), targets identified by BETA only (green bars), or targets identified by LOA and BETA combined (red bars). Error bars indicate mean ± SEM. (e) Violin plots, using the same color coding in (d), of histograms counting the number of HIF-1 target genes showing the differential expression indicated along the abscissa. Target genes in (d) and (e) are separated based on whether their HIF-1 peak is a HOT site or non-HOT site. Solid line indicates median, whereas dotted lines indicate quartiles. ****$p<0.0001$, ANOVA/Kruskal-Wallis Multiple Comparison two-sided test for the indicated comparisons. Nearest neighbors for all HIF-1 binding sites on average only show differential gene expression for non-HOT sites, whereas the average gene neighboring a HOT site shows little differential regulation. By contrast, target genes identified for both HOT and non-HOT sites using BETA only or BETA in combination with LOA show differential gene expression.

Supplementary Figure 6

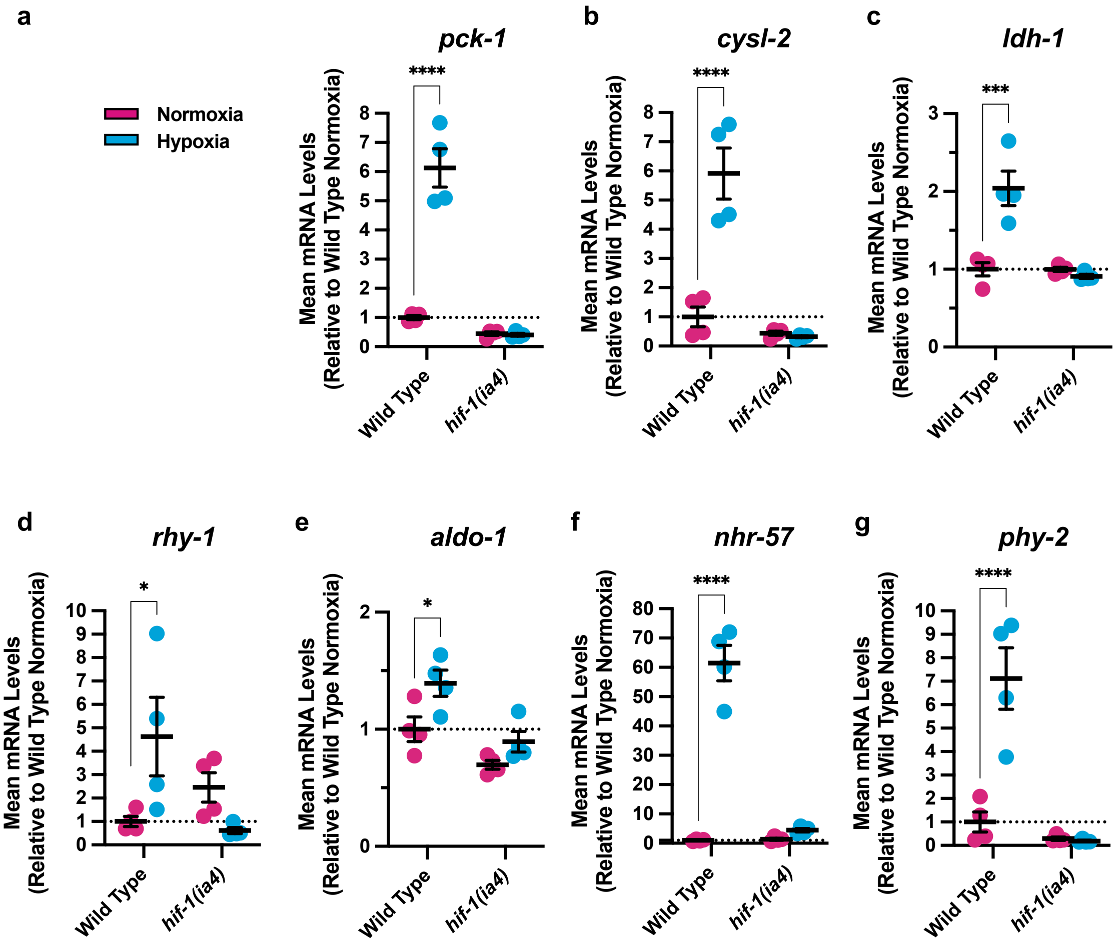

Supplementary Figure 6 Legend. Hypoxia induces the expression of identified HIF-1 targets.

Mean mRNA transcript levels (relative to an actin control) for the given genotypes (indicated along the abscissa) and oxygen conditions (indicated by dot color in the legend, with magenta for normoxia and cyan for 4 hours of 0.5% oxygen hypoxia) as determined by qRT-PCR. The specific gene transcript measured is indicated above each graph: (a) *pck-1*, (b) *cysl-2*, (c) *ldh-1*, (d) *rhy-1*, (e) *aldo-1*, (f) *nhr-57*, and (g) *phy-2*. Data values are normalized to the wild-type normoxic value, which is indicated by a horizontal dotted line. For all graphs, error bars indicate mean ± SEM. ****$p<0.0001$, ***$p<0.001$, **$p<0.01$, *$p<0.05$ ANOVA/Sidak's multiple comparison two-sided test between oxygen conditions. Four biological replicates were analyzed for each gene.

Supplementary Figure 7

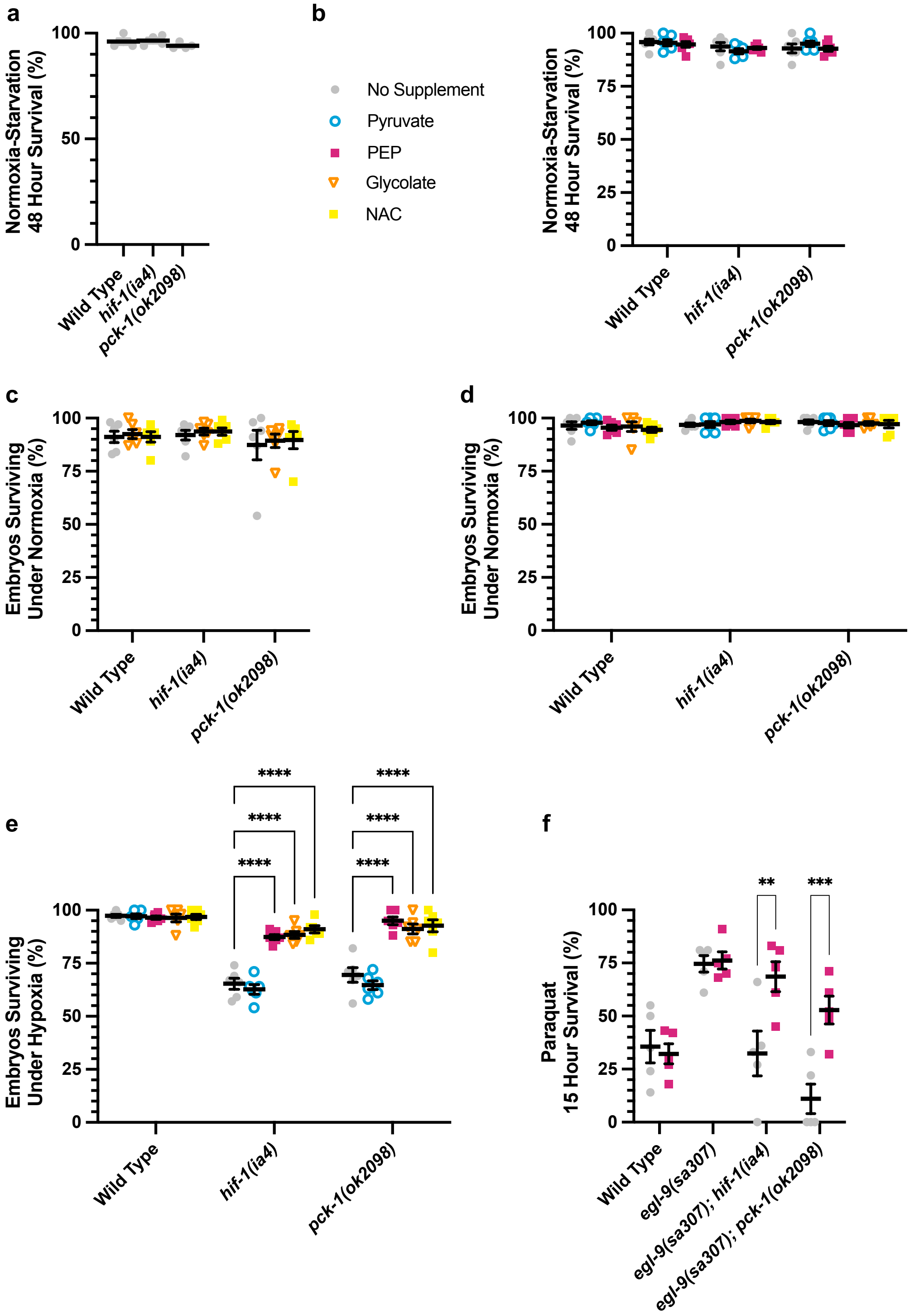

Supplementary Figure 7 Legend. PCK-1 and PEP are required for hypoxia and oxidative stress survival.

(a) Percent of L4-stage animals, grown on fixed *E. coli*, to survive liquid culture without food but with normal oxygen levels (48 hours at 25 °C, with 24 hour recovery at 20°C). (b,c) Percent of embryos, obtained from animals grown on fixed *E. coli*, to survive 24 hours of normoxia at 25°C, with 24 hour recovery at 20°C. Colored symbols (applicable for symbol shapes and colors in all graphs in the figure) indicate the presence of either the indicated metabolite or antioxidant supplement. (d,e) Percent of embryos, obtained from animals grown on live *E. coli*, to survive either (d) 24 hours of normoxia or (e) 24 hours of hypoxia at 25°C, with 24 hour recovery at 20 °C. (f) Percent of L4-stage animals to survive paraquat (200 mM, 15 hours, 20 °C, on plates with food). Error bars indicate mean ± SEM. ****$p<0.0001$, ***$p<0.001$, **$p<0.01$, *$p<0.05$ ANOVA/Sidak's multiple comparison two-sided test between each supplement and the no-supplement control. Data for each column in (a) represents 4 biological replicates, (b-e) represents 6 biologial replicates, and (f) represents 5 biological replicates, typically 20-100 animals per replicate.

Supplementary Figure 8

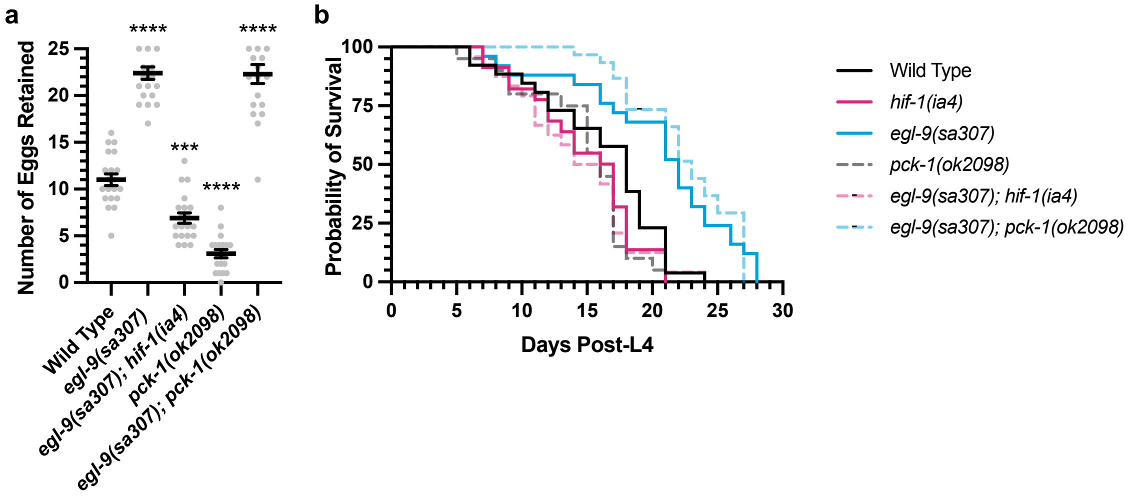

c

condition	chi	pvalue	FDR
Wild Type v.s. *hif-1(ia4)*	2.04	0.1535	0.7674
Wild Type v.s. *egl-9(sa307)*	12.65	0.0004	0.0019
Wild Type v.s. *egl-9(sa307); hif-1(ia4)*	2.04	0.1531	0.7655
Wild Type v.s. *egl-9(sa307); pck-1(ok2098)*	21.51	0.0000035	0.000018
Wild Type v.s. *pck-1(ok2098)*	1.09	0.2966	1
egl-9(sa307) v.s. *egl-9(sa307); pck-1(ok2098)*	0.27	0.605	1

condition	Censored	Deaths	Median Survival (Days)
Wild Type	4	26	18
hif-1(ia4)	8	22	17
egl-9(sa307)	5	25	22
pck-1(ok2098)	0	20	16
egl-9(sa307); hif-1(ia4)	6	24	15
egl-9(sa307); pck-1(ok2098)	12	18	23

Supplementary Figure 8 Legend. PCK-1 is not required for the egg laying or lifespan phenotypes of *egl-9* mutants.

(a) Average number of unlaid eggs *in utero* per animal for the indicated genotype (n=40-55 animals) under normoxia. ****$p<0.0001$, ***$p<0.001$, ANOVA/Dunnett's multiple comparison two-sided test to wild type control. Error bars indicate mean ± SEM. Results from 20 animals for each genotype are shown. (b) Kaplan-Meier survival curves for animals of the indicated age and for the indicated genotypes. (c) P-values and adjusted FDR values as indicated using the Log-rank test.

Supplementary Figure 9

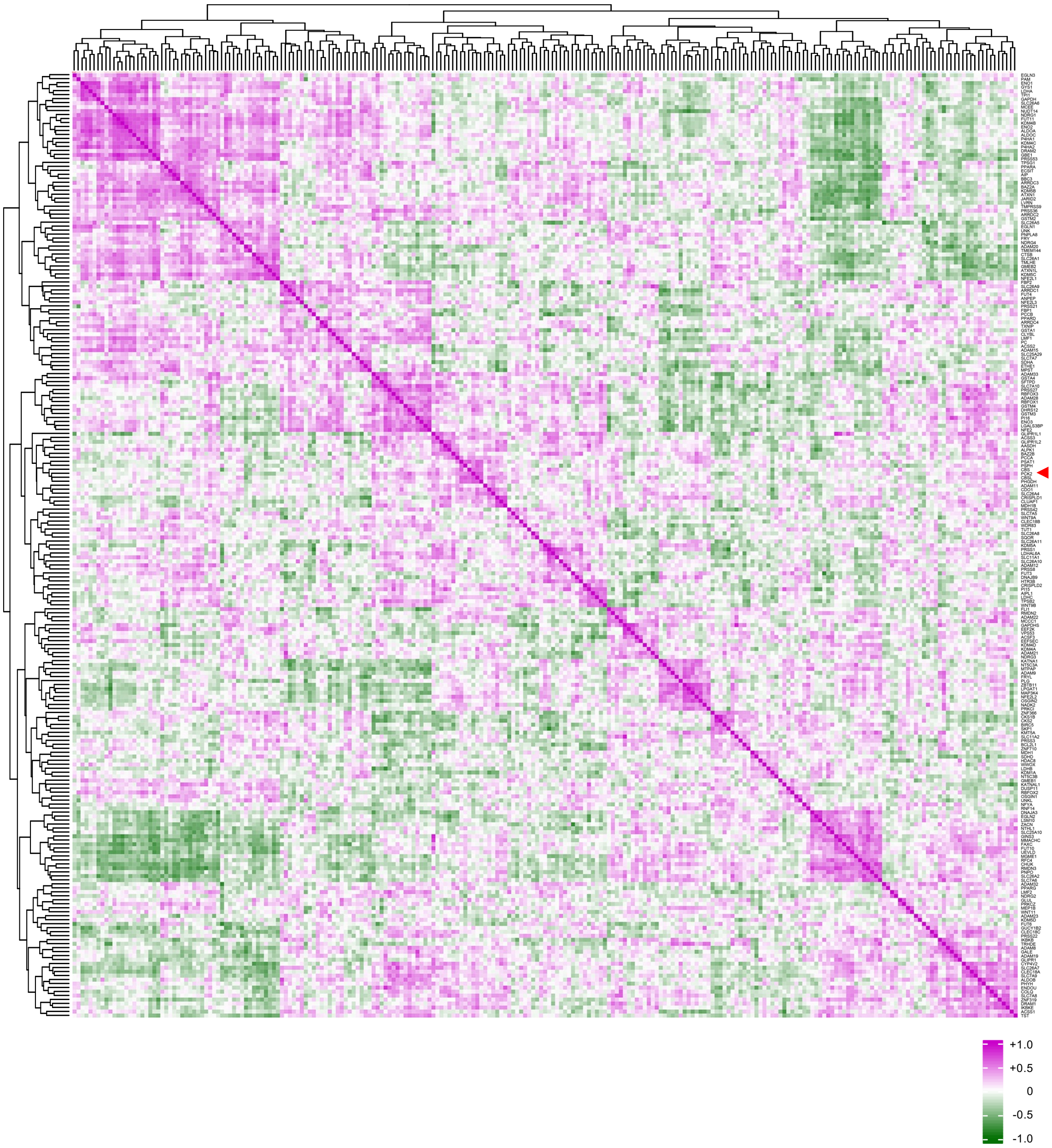

Supplementary Figure 9 Legend. Correlation of differential gene expression for human orthologs of HIF-1 direct targets.

Heat map of mRNA expression correlation across 32 independent experiments involving HIF1A activation. Correlation scores are mapped for individual pairs of human orthologs of the *C. elegans* HIF-1 direct targets identified in this study. Only genes showing expression in at least 15 experiments are shown. Genes (shown on both the X and Y axis) with similar patterns of expression correlation (indicated by magenta) with other genes are hierarchically clustered. Anti-correlation (indicated by green) is also shown. The red arrowhead points to PCK2, the *pck-1* ortholog.
