# Supplementary material for: The hypoxia response pathway promotes PEP carboxykinase and gluconeogenesis in C. elegans
Source: Nat Commun. 2022 Oct 18;13:6168. doi: 10.1038/s41467-022-33849-x (PMC9579151; doi:10.1038/s41467-022-33849-x)
Supplement: Supplementary file 3 — Description of Additional Supplementary Files [file 41467_2022_33849_MOESM3_ESM.pdf]

DESCRIPTION OF ADDITIONAL SUPPLEMENTARY FILES

Title: The Hypoxia Response Pathway Promotes PEP Carboxykinase And Gluconeogenesis In *C. elegans*

Authors: Mehul Vora, Stephanie M. Pyonteck, Tatiana Popovitchenko, Tarmie L. Matlack, Aparna Prashar, Nanci S. Kane, John Favate, Premal Shah, and Christopher Rongo

File Name: Supplementary Data 1
Description: All HIF-1 binding sites identified by ChIP-seq. Includes genome location information and signal value for each site.

File Name: Supplementary Data 2
Description: Differentially expressed genes regulated by HIF-1 and identified by RNA-seq. Includes individual worksheets for each of the 4 genotypic comparisons, as well as for both the upregulated and downregulated genes that lie in the intersection of all 4 of those comparisons. Each gene is represented with WormBase Gene ID, log2 fold change, P-value, and FDR (Benjamini-Hochberg used to adjust the P-values for multiple comparisons).

File Name: Supplementary Data 3
Description: Direct targets of HIF-1 identified by BETA analysis. Includes a worksheet containing the genomic location of each HIF-1 binding site shown by BETA to regulate a nearby gene (indicated by Refseq name), as well as the number of HIF-1 binding sites associated with that specific gene. Also includes a worksheet containing all the direct target genes regulated by one or more nearby HIF-1 binding sites, including the number of associated HIF-1 binding sites and the log2 fold change in expression in *egl-9(sa307)* mutants relative to wild type.

File Name: Supplementary Data 4
Description: Differential metabolite levels regulated by HIF-1 and identified by metabolomics analysis of *egl-9(sa307)* mutants relative to wild type. Includes a worksheet that explains the table terminology, a worksheet containing the raw count data (OrigScale), a worksheet containing rescaled data to set the median equal to 1 (ScaledImpData), a worksheet containing scaled data normalized to total protein per sample (ProNormImpData), and a worksheet containing log2 fold changes between genotypes for each metabolite, associated statistics, and heat map information for significant differences (Pathway Heat Map). The q-values represent FDR, with Benjamini-Hochberg used to adjust the P-values for multiple comparisons.

File Name: Supplementary Data 5
Description: Putative human orthologs of *C. elegans* HIF-1 direct target genes as identified by Ortholist. Each ortholog is represented by an Ensembl ID and HGNC symbol, as well as the WormBase ID, common name, and locus ID of the corresponding *C. elegans* gene.

File Name: Supplementary Data 6
Description: Primers used to analyze target gene expression by qRT-PCR. Forward and reverse sequence are shown, as well as the indicated target being measured.
