# Supplementary material for: The hypoxia response pathway promotes PEP carboxykinase and gluconeogenesis in C. elegans
Source: Nat Commun. 2022 Oct 18;13:6168. doi: 10.1038/s41467-022-33849-x (PMC9579151; doi:10.1038/s41467-022-33849-x)
Supplement: Supplementary file 10 — Reporting Summary [file 41467_2022_33849_MOESM10_ESM.pdf]

nature portfolio

Corresponding author(s): Christopher Rongo

Last updated by author(s): Sep 14, 2022

Reporting Summary

Nature Portfolio wishes to improve the reproducibility of the work that we publish. This form provides structure for consistency and transparency in reporting. For further information on Nature Portfolio policies, see our Editorial Policies and the Editorial Policy Checklist.

Statistics

For all statistical analyses, confirm that the following items are present in the figure legend, table legend, main text, or Methods section.

n/a	Confirmed	
☐	☒	The exact sample size (*n*) for each experimental group/condition, given as a discrete number and unit of measurement
☐	☒	A statement on whether measurements were taken from distinct samples or whether the same sample was measured repeatedly
☐	☒	The statistical test(s) used AND whether they are one- or two-sided<br>*Only common tests should be described solely by name; describe more complex techniques in the Methods section.*
☒	☐	A description of all covariates tested
☐	☒	A description of any assumptions or corrections, such as tests of normality and adjustment for multiple comparisons
☐	☒	A full description of the statistical parameters including central tendency (e.g. means) or other basic estimates (e.g. regression coefficient) AND variation (e.g. standard deviation) or associated estimates of uncertainty (e.g. confidence intervals)
☐	☒	For null hypothesis testing, the test statistic (e.g. F, t, r) with confidence intervals, effect sizes, degrees of freedom and P value noted<br>*Give P values as exact values whenever suitable.*
☒	☐	For Bayesian analysis, information on the choice of priors and Markov chain Monte Carlo settings
☒	☐	For hierarchical and complex designs, identification of the appropriate level for tests and full reporting of outcomes
☒	☐	Estimates of effect sizes (e.g. Cohen's *d*, Pearson's *r*), indicating how they were calculated

Our web collection on statistics for biologists contains articles on many of the points above.

Software and code

Policy information about availability of computer code

Data collection	Microscope images were collected on a CCD camera and digitized using iVision software v4.1 (Biovision Technologies). ChIP-seq sequencing data was collected using an Illumina HiSequation 2000/2500/4000. RNA-seq sequencing data was collected using an Illumina HiSeq2000/2500.
Data analysis	Simple calculations were done in MS Excel v16.64. TIFF files of images were analyzed using open source software (Fiji/ImageJ 2.1.0/1.53c). Quantitative RT-PCR data was analyzed using GraphPad Prism v9.3.0. ChIP-seq reads were aligned to the genome (WS245) using the Burrows-Wheeler Aligner. Peaks of reads were called using a custom SPP ChIP-seq processing pipeline standard for the modERN/ModENCODE public consortium. A broad outline of the modERN/ENCODE approach can be obtained here: https://www.encodeproject.org/pipelines/ENCPL631XPY/. Analysis tools can be obtained at GitHub: https://github.com/ENCODE-DCC/chip-seq-pipeline2/releases/tag/v1.3.5.1. RNA-seq reads were mapped to the genome and counted using STAR 2.5.1a. Normalization and statistical analysis were performed using EdgeR. Metabolomics data was extracted and peak-identified using custom software (Metabolon). Statistical analysis was performed using R in ArrayStudio. Motif enrichment was performed with MEME-Chip . v5.4.1. Data for phenotypic analysis (e.g., survival assays, lifespan assays, egg retention assays, qRT-PCR measurements, individual animal fluorescence measurements) were analyzed using GraphPad Prism 9.3.0. Human transcriptomics meta-analysis was performed using R scripts ggpubR() and pheatmap(); The code used to generate these figures can be found at https://github.com/shahlab/hypoxia-multiomics. Specific statistical tests are indicated in the figure legends, including P values. All tests were two-sided unless otherwise indicated. Data normality was tested using Kolmogorov-Smirnov and adjusted for multiple comparisons as indicated in the figure legends.

For manuscripts utilizing custom algorithms or software that are central to the research but not yet described in published literature, software must be made available to editors and reviewers. We strongly encourage code deposition in a community repository (e.g. GitHub). See the Nature Portfolio guidelines for submitting code & software for further information.

Data

Policy information about availability of data

All manuscripts must include a data availability statement. This statement should provide the following information, where applicable:

- Accession codes, unique identifiers, or web links for publicly available datasets
- A description of any restrictions on data availability
- For clinical datasets or third party data, please ensure that the statement adheres to our policy

Data supporting the findings of this study are in publicly available repositories. ChIP-seq data sets are available at NIH/NCBI GEO through accession number GSE7173333. Files can be directly accessed at the web link https://www.ncbi.nlm.nih.gov/geo/query/acc.cgi?acc=GSE173333. The input file for OR3349 was GSM5266000, whereas the two replicates for OR3349 were GSM5266001 and GSM5266002. The input file for OR3350 was GSM5266003, whereas the two replicates for OR3350 were GSM5266004 and GSM5266005. BED files can be obtained and viewed using the UCSC browser at https://tinyurl.com/4pxu6vvv. Files for RNA-seq data sets are available at NIH/NCBI GEO through accession number GSE173581. Files can be directly accessed at the web link https://www.ncbi.nlm.nih.gov/geo/query/acc.cgi?acc=GSE173580. The files GSM5271168, GSM5271169, GSM5271176, and GSM527117 contain data for four independent biological replicates for N2 wild-type nematodes. The files GSM5271170, GSM5271171, GSM5271178, and GSM5271179 contain data for four independent biological replicates for hif-1(ia4) mutant nematodes. The files GSM5271172, GSM5271173, GSM5271180, and GSM5271181 contain data for four independent biological replicates for egl-9(sa307) mutant nematodes. The files GSM5271174, GSM5271175, GSM5271182, and GSM5271183 contain data for four independent biological replicates for egl-9(sa307) hif-1(ia4) mutant nematodes. The files GSM5271184, GSM5271185, GSM5271186, and GSM5271187 contain data for four independent biological replicates for OR3350 nematodes. A broad outline of the modERN/ENCODE approach can be obtained here: https://www.encodeproject.org/pipelines/ENCPL631XPY/. Analysis tools can be obtained at GitHub: https://github.com/ENCODE-DCC/chip-seq-pipeline2/releases/tag/v1.3.5.1. C. elegans ModENCODE datasets can be obtained at http://www.modencode.org/publications/worm_2010pubs/index.shtml.
Other source data are provided online as a single Source Data with this paper.

Field-specific reporting

Please select the one below that is the best fit for your research. If you are not sure, read the appropriate sections before making your selection.

☒ Life sciences ☐ Behavioural & social sciences ☐ Ecological, evolutionary & environmental sciences

For a reference copy of the document with all sections, see nature.com/documents/nr-reporting-summary-flat.pdf

Life sciences study design

All studies must disclose on these points even when the disclosure is negative.

Sample size	Statistical power analysis was performed for sample size estimations using G*Power based on our own preliminary analysis of mutants at the beginning of the study (or from previous measurements of the mutants as published), where effect size compared to wild type has been large (d=2.3-4.8) depending on the phenotypic assay. With an alpha=0.5 and power=0.95, and assuming a modest effect size (d=0.8), we estimated about 40 animals per genotype and condition. Replicate numbers for ChIP-seq, RNA-seq, and metabolomics were chosen based on standard deviation and effect size determined from our previous preliminary data, balanced with the cost of these assays.
Data exclusions	For lifespan assays, animals that showed defects due to aberrant vulval development or egg laying (e.g. bursting at the vulva, bagging, etc.) or desiccated on the side of the dish were censored at the time of their demise. Specific numbers of counted and censored animals are included in the lifespan figure. These are standard procedures for C. elegans lifespan assays, with criteria that were pre-established prior to the experiment.
Replication	Data for phenotypic analysis (e.g., survival assays, lifespan assays, egg retention assays, individual animal fluorescence measurements) were analyzed using at least 3-5 independent biological replicates, with typically 50 animals assayed per replicate. For data shown, all replicates behaved consistently and reproduced the same finding. QRT-PCR analysis was replicated 2-6 times depending on the specific experiment, with all replicates behaving consistently and reproducing the same finding. RNA-seq was analyzed with 4 biological replicates, all of which behaved consistently and reproduced the same finding. ChIP-seq was analyzed with 2 biological replicates, both of which behaved consistently and reproduced the same finding. Metabolomics was analyzed with 9 biological replicates, all of which behaved consistently and reproduced the same finding.
Randomization	Individual nematodes were sorted into groups to individual Petri dishes based on genotype (assayed by PCR or sequencing). Plates containing nematodes of a given genotype were then picked and exposed to treatment (if applicable) with first examining the animals at the microscopic level. Individual nematodes from each genotype and treatment were then picked randomly from these Petri plates.
Blinding	In all experiments, researchers were blinded to genotype or experimental treatment during the data collection and analysis. The egg laying defects for egl-9 mutants made blinding impossible for scoring egg laying (Sup Fig 1h, Sup Fig 8a), as these animals retain eggs, which is easily visible to any researcher doing the scoring and analysis.

Reporting for specific materials, systems and methods

We require information from authors about some types of materials, experimental systems and methods used in many studies. Here, indicate whether each material, system or method listed is relevant to your study. If you are not sure if a list item applies to your research, read the appropriate section before selecting a response.

Materials & experimental systems

n/a	Involved in the study
☐	☒ Antibodies
☒	☐ Eukaryotic cell lines
☒	☐ Palaeontology and archaeology
☐	☒ Animals and other organisms
☒	☐ Human research participants
☒	☐ Clinical data
☒	☐ Dual use research of concern

Methods

n/a	Involved in the study
☐	☒ ChIP-seq
☒	☐ Flow cytometry
☒	☐ MRI-based neuroimaging

Antibodies

Antibodies used

A non-commercial anti-GFP antibody (the poly-clonal goat IgG anti-GFP described below) developed by Tony Hyman and Kevin White for the ModENCODE/modERN consortium was used for the ChIP-seq analysis. Since this antibody was not developed commercially, there is no supplier name, catalog number, clone name, or lot number.

Validation

This poly-clonal goat IgG anti-GFP antibody was validated by Western blot of immunoprecipitated material from transgenic animals expressing AMA-1::GFP: see reference Zhong, M. et al. Genome-wide identification of binding sites defines distinct functions for Caenorhabditis elegans PHA-4/FOXA in development and environmental response. PLoS Genet 6, e1000848, doi:10.1371/journal.pgen.1000848 (2010). Comparison were made to control immunoprecipitations using mouse and goat IgG, as well as comparisons to input to establish background. Similarity to native profiles were determined by comparing ChIP-seq datasets between GFP-tagged AMA-1 (Pol II) and the native protein precipitated by another antibody directly raised against native AMA-1, resulting in correlations of 0.93 and 0.95 for two biological replicates.

Animals and other organisms

Policy information about studies involving animals; ARRIVE guidelines recommended for reporting animal research

Laboratory animals

Laboratory strains of C. elegans, all of which are variants of the original N2 strain. Only hermaphrodites were examined, typically at the L4 stage, although some lifespan analysis was conducted on animals that ranged from embryo all the way to day 20 animals. Only hermaphrodites were analyzed.

Wild animals

No wild animals were used in the study.

Field-collected samples

No field collected samples were used in the study.

Ethics oversight

Not applicable for C. elegans nematodes.

Note that full information on the approval of the study protocol must also be provided in the manuscript.

ChIP-seq

Data deposition

☒ Confirm that both raw and final processed data have been deposited in a public database such as GEO.

☒ Confirm that you have deposited or provided access to graph files (e.g. BED files) for the called peaks.

Data access links
May remain private before publication.

ChIP-seq files can be directly accessed at the web link https://www.ncbi.nlm.nih.gov/geo/query/acc.cgi?acc=GSE173333, using token ajudwmgihrwxhyf.

Files in database submission

The ChIP-seq input file for OR3349 was GSM5266000, whereas the two replicates for OR3349 were GSM5266001 and GSM5266002. The ChIP-seq input file for OR3350 was GSM5266003, whereas the two replicates for OR3350 were GSM5266004 and GSM5266005.

Genome browser session
(e.g. UCSC)

ChIP-seq BED files can be obtained and viewed using the UCSC browser at https://tinyurl.com/4pxu6vvv.

Methodology

Replicates

For ChIP-seq studies, two independent biological replicates were performed for each genotype. Only peaks that showed an irreproducibility discovery rate (IDR) of 0.1% between the two replicates were used to generate the final peak sets.

Sequencing depth	Sequencing was performed on the Illumina HiSequation 2000/2500/4000, resulting in a range of 6.5-14.1 M single end, 50-bp reads for input and both replicates for OR3349 and OR3350.
Antibodies	A non-commercial anti-GFP antibody developed by Tony Hyman and Kevin White for the ModENCODE/modERN consortium was used for the ChIP-seq analysis. This poly-clonal goat IgG anti-GFP antibody was validated by Western blot of immunoprecipitated material from transgenic animals expressing AMA-1::GFP: see reference Zhong, M. et al. Genome-wide identification of binding sites defines distinct functions for Caenorhabditis elegans PHA-4/FOXA in development and environmental response. PLoS Genet 6, e1000848, doi:10.1371/journal.pgen.1000848 (2010). Comparison were made to control immunoprecipitations using mouse and goat IgG, as well as comparisons to input to establish background. Similarity to native profiles were determined by comparing ChIP-seq datasets between GFP-tagged AMA-1 (Pol II) and the native protein precipitated by another antibody directly raised against native AMA-1, resulting in correlations of 0.93 and 0.95 for two biological replicates.
Peak calling parameters	The Illumina sequencing data were aligned to the reference genome using the Burrows–Wheeler Aligner (BWA). Data were aligned to genome version WS245. A range of 40.4%-85% of reads aligned. Peak regions significantly enriched in aligned reads were called by the SPP ChIP-seq processing pipeline standard for modERN/ModENCODE. Peaks above an irreproducibility discovery rate (IDR) of 0.1% were used to generate final peak sets. Lowest enrichment values for OR3349 and OR3350 were 93.8 and 50.8, respectively. A broad outline of the modERN/ENCODE approach can be obtained here: https://www.encodeproject.org/pipelines/ENCPL631XPY/. Analysis tools can be obtained at GitHub: https://github.com/ENCODE-DCC/chip-seq-pipeline2/releases/tag/v1.3.5.1.
Data quality	Phred scores ranged from 32-38 out to at least 47 bases for all samples. Peaks above an irreproducibility discovery rate (IDR) of 0.1% were used to generate final peak sets. Lowest enrichment values for OR3349 and OR3350 were 93.8 and 50.8, respectively.
Software	Peak regions significantly enriched in aligned reads were called by the SPP ChIP-seq processing pipeline standard for modERN/ModENCODE. A broad outline of the modERN/ENCODE approach can be obtained here: https://www.encodeproject.org/pipelines/ENCPL631XPY/. Analysis tools can be obtained at GitHub: https://github.com/ENCODE-DCC/chip-seq-pipeline2/releases/tag/v1.3.5.1.
